# Implantable and Injectable Biomaterial Scaffolds for Cancer Immunotherapy

**DOI:** 10.3389/fbioe.2020.612950

**Published:** 2020-11-30

**Authors:** Jie Li, Yiqian Luo, Baoqin Li, Yuanliang Xia, Hengyi Wang, Changfeng Fu

**Affiliations:** Department of Spine Surgery, The First Hospital of Jilin University, Changchun, China

**Keywords:** implantable scaffold, injectable scaffold, hydrogel, biomaterial, immunotherapy

## Abstract

Cancer immunotherapy has become an emerging strategy recently producing durable immune responses in patients with varieties of malignant tumors. However, the main limitation for the broad application of immunotherapies still to reduce side effects by controlling and regulating the immune system. In order to improve both efficacy and safety, biomaterials have been applied to immunotherapies for the specific modulation of immune cells and the immunosuppressive tumor microenvironment. Recently, researchers have constantly developed biomaterials with new structures, properties and functions. This review provides the most recent advances in the delivery strategies of immunotherapies based on localized biomaterials, focusing on the implantable and injectable biomaterial scaffolds. Finally, the challenges and prospects of applying implantable and injectable biomaterial scaffolds in the development of future cancer immunotherapies are discussed.

## Introduction

Immunotherapy has revolutionized the paradigm of cancer treatment, aiming to stimulate inherent immunological systems to indirectly attack tumor cells (Yang, 2015). Cancer immunotherapy has fewer off-target effects compared with chemotherapy or other therapeutic methods that directly eliminate tumor cells (Chen et al., 2020; Feng et al., 2019b; Riley et al., 2019).

Immunotherapy has five main classes: immune checkpoint blockade (ICB) therapy, lymphocyte-promoting cytokine therapy, chimeric antigen receptor T-cell (CAR-T) therapy, agonistic antibodies, and tumor vaccines (Zhao et al., 2019). Among these, ICB therapy is the most comprehensively studied class of immunotherapy till now (López-Soto et al., 2017). The blocking of cytotoxic T-lymphocyte-associated antigen 4 (CTLA-4) and programmed cell death protein 1 (PD-1/PD-L1) pathways have been the most commonly used checkpoint inhibition strategies (Chen et al., 2019). The clinical therapeutic effect and application of PD-1/PD-L1 and CTLA4 checkpoint blockade methods have significantly increased in the last few years owing to the excellent clinical efficacy (Postow et al., 2015). In the aspect of biology, CTLA-4 is only expressed on T cells to modulate the amplitude of T cells activity at early stage. However, the exact cellular mechanism of the function of CTLA-4 remains unclear. The current conclusion is that CTLA-4 and CD28 competitively bind to CD80 and CD86 ligand to dampen the activation of T cells, so as to promote tumor progression. Though CTLA-4 is induced by activated CD8+ T cells, the main function of it may realize by downmodulate the activity of helper T cell and enhance the regulatory of regulatory T cells (Tregs). The major faction of PD-1 is to restrict the activity of T cells in tumor sites at the time of an immune response. The expression of PD-1 is induced when T cells are activated, which enables T cells to recognize abnormal cells. However, tumor cells can adaptively express of programmed death ligand 1 (PD-L1), a binder of PD-1 to inhibit T cells activity, to avoid being recognized and killed by T cells. Similarly, PD-1 is also highly expressed on Tregs to improve their proliferation, leading to further immunosuppression in the tumor microenvironment. Therefore, blocking the PD-1 pathway can also improve the effect of immunotherapy by inhibiting the proliferation of Tregs (Lohmueller and Finn, 2017). However, the side effects in organs after the systemic administration of checkpoint inhibitors remains the main limitation for clinical use. In addition, the immunosuppressive tumor microenvironment can result in no response of patients to the treatment (Wilky, 2019). Cytokine therapy was the first clinically used immunotherapy on account of the approval of recombinant IFNα therapies in 1986 (Riley et al., 2019). Interferons can not only bind to the receptors on the surface of innate immunity cells to activate natural killer (NK) cells and macrophages, but up-regulate the major histocompatibility complex (MHC) of T cells to improve their antigen presentation ability and activate their adaptive immunity. Interleukins can mediate the activation and proliferation of CD4+ T cells, CD8+ T cells and NK cells. Interferons, interleukins and granulocyte macrophage colony-stimulating factor (GM-CSF) are three commonly used types of cytokines (Koshy and Mooney, 2016). GM-CSF can promote the ability of dendritic cells (DCs) to process and present tumor antigens, so as to induce antitumor cytotoxic T lymphocytes response. Besides, GM-CSF can also promote the homeostasis of T cells to increase the survival of them. Though cytokine therapy has made great progress in the treatment of multiple malignant tumors, several limitations still hinder its development. The transitory half-life of injected cytokines results in bolus injections in clinical treatment, leading to vascular leakage and cytokine release syndrome of patients (Rooney and Sauer, 2018). Adoptive T cell therapy involves the modification of a variety of cells, including DCs, NK cells and T cells. Among them, T cells are the first choice for adoptive T cell therapy owing to their endogenous ability of identifying and diminishing tumor cells through releasing perforin, granzyme and various of cytokines. T cells collected from patients are then engineered to express chimeric antigen receptors that are antigen-specific to tumor cells (Wei et al., 2019). Then, the engineered cells are injected back into patients to target the antigen of tumor cells and kill them (Wang et al., 2017). To overcome the downregulation of the expression of MHC class I molecules in tumor cells and promote the infiltration of programmed T cells to tumor sites, artificial chimeric antigen receptors (CAR) have been developed to equip T cells isolated from patients with the capacity of recognizing and targeting tumor cell surface antigens. Nevertheless, the cytokine release syndrome and low response to solid tumors are the two key challenges of CAR-T therapy (Majzner and Mackall, 2018). The temporary efficacy of the treatment towards solid tumors is on account of the immunosuppressive tumor microenvironment and the selective escape of tumor cells from immune detection (Neelapu et al., 2018). The methods to promote the viability and activity of exogenous T cells are described below. Agonistic antibodies can connect to receptors on the surface of T cells, and trigger intracellular signaling pathways, leading to the survival and growth of T cells (Shi et al., 2018). T cell receptors that are most commonly targeted receptors, which consist of co-stimulatory receptors (CD28) and the tumor necrosis factor receptor (TNFR) family (Walsh et al., 2015). At present, agonistic antibodies are still in the initial stage of progression, and many deficiencies remain to be solved. For example, agonistic antibodies have dose-dependent toxicities, just as those for cytokines, since these could mediate the activity in undesired types of immune cells and attack on healthy cells (Zippelius et al., 2015). Cancer vaccines consist of tumor cell lysate, nucleic acids, DCs, or neo-antigens. DCs vaccines are the most widely studied class among them (Lohmueller and Finn, 2017). DCs extracted are engineered to produce tumor-associated antigens, activating T cells to directly kill tumor cells (Sahin and Türeci, 2018). However, the limited therapeutic effect, the uncertain application dose, and the complicated manufacturing processes of cancer vaccines lead to insufficient immune responses and poor anti-tumor effect (Aurisicchio et al., 2018).

New approaches for cancer immunotherapy are needed to promote the therapeutic potential of therapeutic payloads through a safer and more controlled manner. A large and growing body of studies have demonstrated the synergistic effects of biomaterials combined with cancer immunotherapy direct the path to address these limitations (Guo et al., 2020; Sang et al., 2019; Zhang et al., 2020). Lately, a variety of biomaterials, such as nanoparticles, implantable biomaterial scaffolds and injectable biomaterial scaffolds, have been introduced to promote immune response and improve the anti-tumor effect (Feng et al., 2020; Li S et al., 2018; Li et al., 2019; Wang et al., 2020; Xie et al., 2018). Improved delivery technologies using these materials can induce systemic immune therapeutic responses, while avoiding systemic toxicity (Chen et al., 2019; Ding et al., 2019c; Qiu et al., 2020).

**FIGURE 1:**
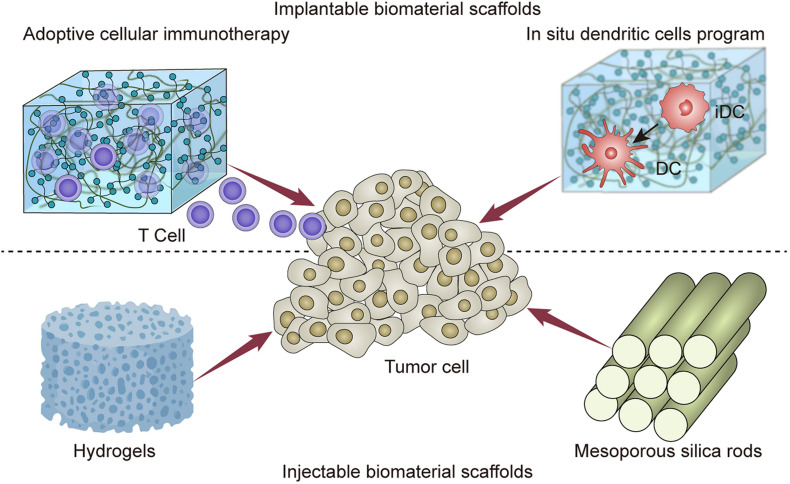
Implantableand injectable biomaterial scaffolds for cancer immunotherapy.

In the present review, the investigators focused on the advances in implantable and injectable scaffolds in achieving spatial and temporal controlled delivery (Table 1). These positionable scaffolds presented great potential in the delivery of immune agents, and the induction of systemic immune response (Figure 1). It is hoped that this review can assist medical workers for comprehensively understanding the latest progress and future prospects of the combination of immunotherapy with biomaterials.

**TABLE 1:** Implantable and injectable biomaterial scaffolds for cancer immunotherapy.

	Material type	Payload	Results	References
Implantable scaffolds	Alginate scaffold	CAR-T cells	Proliferate T cells and reduce the unresectable or incompletely resected tumors.	Stephan et al., 2015
	Alginate scaffold	CAR-T cells, STING agonists	Stimulate systemic immune response to eliminate solid tumors.	Smith et al., 2017
	Hyaluronic acid scaffold	CAR-NK cells	Enhance the expansion, persistency and antitumor efficiency of NK cells.	Ahn et al., 2020
	Collagen and HA cross-linking scaffold	GEM, poly(I:C)	Reduce the tumor-infiltrating MDSCs and increase the number of CD8+ T cells.	Phuengkham et al., 2018
	PLG scaffold	GM-CSF, CpG-ODNs	Recruit, activate and home to lymph nodes of DCs.	Ali et al., 2009
Injectable scaffolds	Alginate hydrogel	GM-CSF	Recruit CD11b+ CD11c+ DCs into the hydrogels.	Verbeke and Mooney, 2015
	Alginate hydrogel	Microparticles, peptide antigens	Recruit and activate immune cells	Verbeke et al., 2017
	Alginate hydrogel	Celecoxib, PD-1 antibody	Regulate the immunosuppressive tumor microenvironment and improve antitumor activities.	Li Y. et al., 2016
	PEGylated poly(L-valine) hydrogel	TCL, poly(I:C)	Enhance the percentage of migratory DCs in tumor-draining lymph nodes and induce cytotoxic T-lymphocyte immune response.	Song et al., 2018
	RADA16 peptide hydrogel	PD-1 antibodies, DCs, TCL	Increase the percentage of CD8+ IFN-γ+ T cells.	Yang et al., 2018
	ROS-degradable hydrogel	GEM, PD-L1 antibody	Achieve obvious tumor suppression effects and induce a T cell immune response.	Wang C. et al., 2018
	D-tetra-peptide hydrogel	OVA, X-ray irradiated E.G7 tumor cells	Induce powerful CD8+ IFN-γ+ T cell immune response.	Luo et al., 2017
	Phospholipid hydrogel	OVA, CpG-ODN	Recruit and activate DCs, induce memory T cells response.	Han et al., 2016
	HA-Tyr hydrogel	IFN-α, sorafenib	Induce apoptosis of tumor cells and the suppress the angiogenesis.	Ueda et al., 2016
	Peptide hydrogel	CDN	Achieve powerful immune memory effect to resist a secondary injection of tumor cells.	Leach et al., 2018
	MSR	OVA, GM-CSF, CpG-ODN	Recruit DCs, increase the systemic TH1 and TH2 serum antibody and cytotoxic T cells.	Kim et al., 2015
	PEI with MSR	E7 peptide	Recruit and activate DCs and the immune response of T cells.	Li A.W. et al., 2018
	PEG, RGD, or RDG modified MSR	None	Increase BMDC activation marker expression and the innate immune cells infiltration.	Li W.A. et al., 2016

## Implantable Biomaterial Scaffolds for Immunotherapy

### Implantable Biomaterial Scaffolds for Adoptive Cellular Immunotherapy

Implantable biomaterial scaffolds preloaded with immune agents, bioactive factors, or cells can be implanted into resected tissue space or a subcutaneous via a small surgical procedure. Immune cells can be recruited into scaffolds and activated for further biological programming with the slow release of immunoregulatory agents (Chew and Danti, 2017; Ding et al., 2019b; Feng et al., 2019a).

A representative research designed macroporous scaffolds from polymerized alginate for stocking, proliferation and dispersing engineered T cells. This approach aimed to manage locally advanced, and unresectable or incompletely resected tumors through situating implants near these. To make the alginate scaffolds, the authors used calcium chloride for the cross-linking agent and round Teflon-coated mold to form 2 mm-thick scaffolds. These scaffolds were frozen and lyophilized to obtain porous matrices. In the mice breast cancer resection model, the proliferation of T cells from the scaffold at the implanted site was 167 times of that injected through conventional delivery modalities, resulting in the reduction rate of postoperative metastasis and recurrence. In addition, in a multifocal ovarian cancer model, the authors demonstrated that T cells from the scaffold triggered the regression, while the injected tumor-reactive lymphocytes had small curative effect (Stephan et al., 2015; Zheng P et al., 2019). In another research, the authors also used alginate scaffolds to deliver chimeric antigen receptor T-cell immunotherapy y (CAR-T) cells, and achieved a good therapeutic effect. In addition, the authors demonstrated that the combination of inducer of interferon genes (STING) agonists with alginate scaffolds stimulated a strong immune response to kill tumor cells unidentified by lymphocytes (Smith et al., 2017; Ding et al., 2019a).

NK cells are able to separate malignant tumor cells from normal cells in an antigen-independent method through identifying the mismatch of inhibitory signaling pathways. This leads to the preference to eliminate stem cell-like tumor cells that have promoted tumorigenic effects, which are incentive to traditional therapies (Guillerey et al., 2016; Tian, 2017; Sanchez-Correa et al., 2019). Due to the poor infiltration of NK cells in the tumor microenvironment, the clinical effects towards solid tumors remain unsatisfactory (Davis et al., 2017; Siegler et al., 2018). In addition, with the deepening of studies on NK cells, other deficiencies in cell enrichment technique, targeting effect, dependency on stimulating cytokines, and tumor elimination ability are emerging (Shimasaki et al., 2020). Therefore, a representative study synthesized a 3D-engineered hyaluronic acid (HA)-based niche for the expansion of NK cells, which was called 3D-ENHANCE. NK cells were loaded with deformed states in this biodegradable and biocompatible polymeric scaffold due to the excellent hydration ability of HA. HA can regulate the proliferation and migration of NK cells as a key member of the extracellular matrix (ECM). Compared with two-dimensional (2D) (petri dish), 3D-ENHANCE promotes the intercellular interaction and cell aggregation of NK cells, leading to the increase in cell proliferation and high cell viability. To study the specific mechanism of 3D-ENHANCE in promoting NK cell interaction and cell aggregation, the authors extracted ribonucleic acid for sequencing and analyzed the transcript information. The results showed that 236 genes were up regulated with the treatment of 3D-ENHANCE. The expressions of CDK6, CCNB1, and CDC20 improved the cell division and proliferation, the expressions of lymphotoxin-alpha, IL-6, and tumor necrosis factor α improved the inflammatory response, while the expressions of IFN-γ and granzyme B enhanced the cytotoxicity of NK cells. Next, the investigators tested the cytotoxicity induced by cultures in 2D and 3D, and it was demonstrated that 3D scaffolds had more powerful tumor killing activity towards NK cells. After the injection of 3D-ENHANCE, which was loaded with zEGFR (epidermal growth factor receptor)-CAR NK cells, the postoperative metastasis and recurrence of MDA-MB-231 model with incomplete resection significantly decreased by implantation. In addition, the survival of mice was extended in the K562 leukemia model after the intravenous injection of engineered NK-92 cells expanded in 3D-ENHANCEs. With the favorable mRNA expression, the increase in cytokine release and tumor-lytic abilities, 3D-ENHANCE can significantly enhance the cell expansion, persistency and antitumor efficiency. Overall, 3D-ENHANCE provides a promising strategy for ex vivo expansion and postsurgical treatment to improve the poor therapeutic effect of NK cells therapy (Ahn et al., 2020).

Implantable biomaterial scaffolds solve some of the limitations of present adoptive cellular immunotherapies (Mouthuy et al., 2016). First, the direct injection of a large number of tumor-reactive lymphocytes for the treatment of solid tumors is commonly non-effective due to the low accumulation and expansion of the lymphocytes at the tumor sites (Rao et al., 2016). The scaffolds can regulate the immunosuppressive tumor microenvironment and continuously disperse the lymphocytes (Bersani et al., 2014). Second, the procedure of adoptive cellular immunotherapy is complex, which can result in the functional exhaustion of cells before reintroduced (Leach et al., 2019). In the biomaterial approach, proliferation and activation factors are loaded into the scaffold. Engineered lymphocytes can immediately eliminate adjacent tumor cells, leading to the minimized side effects and facilitated recovery of patients (Seib et al., 2015). In addition, this platform can not only deliver various kinds of lymphocytes, but also deliver cells that are difficult to proliferate, or that needs a strict microenvironment, such as type 1 T-helper cells or stem cell-like memory T cells (Pelaez et al., 2018).

### Implantable Biomaterial Scaffolds Program Dendritic Cells *in situ*

**FIGURE 2:**
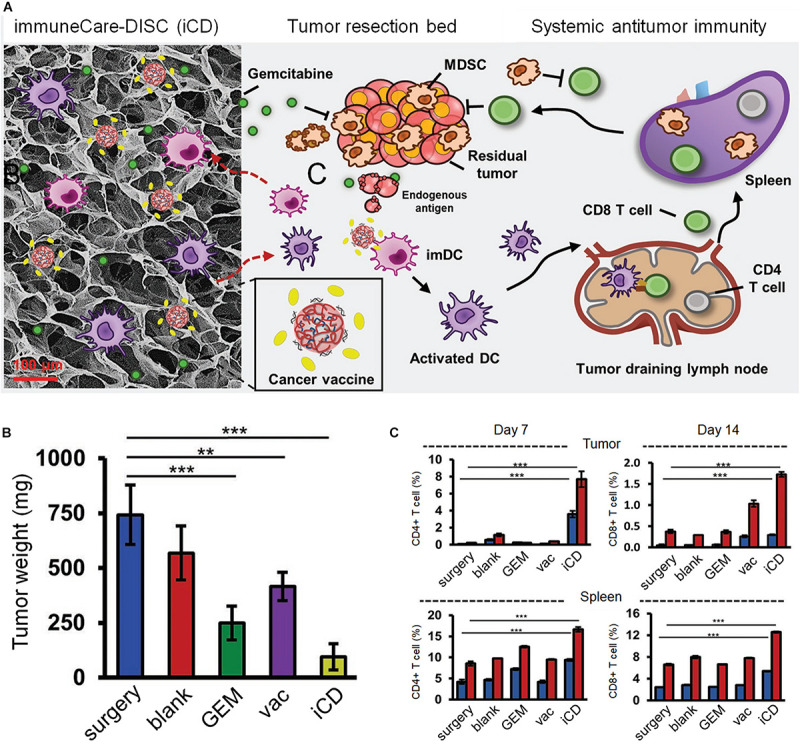
The 3D scaffolds through the crosslinking HA and collagen to deliver GEM and poly(I:C) for postoperative immunotherapy. **(A)** The designed scaffolds carrying GEM, vaccines and TLR3 agonists to promote cancer immunotherapy. **(B)** The weight of recurring tumors on day 14 after incomplete resection. **(C)** The percentage of immune cells at day 7 and 14. The bule represents CD3+ CD8+ T cells and the red represents CD3+ CD4+ T cells. Reproduced with permission from Phuengkham et al. (2018).

Tumor vaccines provide an attractive choice to improve the postoperative survival rate of patients (Butterfield, 2015; Srinivasan et al., 2017). Introducing tumor antigens to DCs has been demonstrated to be an effective kind of strategy in vaccine and immunotherapy (Sabado and Bhardwaj, 2015; Saxena and Bhardwaj, 2018; Wang P. et al., 2018). However, the immunosuppressive tumor microenvironment is the main obstacle for completely eliminating the tumor (Yang et al., 2019). Gemcitabine (GEM) has been demonstrated to be immunological in exhausting MDSCs, which is a critical player in the immunosuppression tumor microenvironment of mice models and patients, resulting in the relief of the immunosuppressive tumor microenvironment (Zhang et al., 2019). The mechanism of by which this effect is achieved is unclear. One possible reason is that GEM can cause a massive efflux MDSCs into the blood and other organs, while another possible reason is that GEM can selectively kill Gr-1+/CD11b+ MDSCs without affecting other immune cells. Further studies are still needed to discover the mechanism and biochemical effects of GEM on MDSCs (Suzuki et al., 2005). A representative research introduced a 3D scaffold by cross-linking collagen and HA to deliver GEM and poly(I:C), which can trigger an intense immune response through stimulating TLR3 in DCs and macrophages. The combination of collagen and HA was demonstrated to promote cell migration and division due to the preeminent biocompatibility and biodegradability. The 3D scaffolds were implanted into a 4T1 local recurrence mice model. The results indicated a significant reduction in tumor-infiltrating MDSCs, and an increased number of CD8+ T cells. In addition, the infiltration of DCs and macrophages in the tumor site and spleen were also markedly increased. The 3D scaffolds can be used as an immune inducing center for the recruitment and education of DCs, and can be expected to provide a choice to prevent postoperative tumors from recurrence and metastasis (Figure 2; Phuengkham et al., 2018).

Another study used porous poly(lactide-co-glycolide) (PLG) scaffold for the continuously delivery of granulocyte-macrophage colony stimulating factor (GM-CSF) or CpG oligodeoxynucleotides (CpG-ODNs) to recruit and induce the proliferation of DCs. The investigators used two methods to recruit and release DCs. The first method took advantage of the release of GM-CSF alone to recruit DCs into the scaffolds. The results revealed that the DCs were subsequently recruited and trapped in the scaffolds. The DCs could be activated and disperse only when the GM-CSF levels fall, indicating that the specific concentration and duration of GM-CSF can profoundly affect the effects of the treatment. This method created a physical environment, and provided stimulatory signals to DCs for over two weeks. Another method developed a continuous process to shuttle DCs. The authors first used GM-CSF to recruit DCs into the scaffolds, and then used the subsequent release of CpG-ODN to activate the resident DCs. The results demonstrated that the presentation of CpG-ODN significantly enhanced the expansion of activated DCs, and the percentage of programmed DCs that migrated to the lymph nodes, indicating that the mimicking aspects of infection can effectively impact the recruitment, activation and homing to the lymph nodes of DCs. Optimistically, in the preclinical melanoma mice model, tumors completely subsided in 47% of mice (Ali et al., 2009; Sterner et al., 2019).

In order to realize to recruit, activate and disperse the DCs, these studies were steered by the cumbersome steps and cost of cell manipulation and transplantation ex vivo to program DCs in situ. This implantable material approach could be used as a substitution to present cancer vaccine strategies, or in combination with other methods. Furthermore, this research shows powerful new applications of polymeric biomaterials, which could be applied in a various of diseases through in-situ programming or reprogramming of host cells. Overall, the thought of implantable biomaterials for DCs in situ program may offer a new approach for polymer therapy, and a promising choice to cell therapies that rely on ex vivo cell manipulation.

In conclusion, implantable biomaterial scaffolds can remain at the site of implantation for a long time to maintain antigen presentation, control cell transport, recruit immune cells, and perform several other functions (Chung et al., 2017). However, the drawbacks of implantable scaffolds are also obvious. Invasive surgery needs to implant the scaffolds in or close to the tumor site (Youssef et al., 2017). Moreover, the scaffolds cannot be administered into inaccessible sites or volume-sensitive regions during the surgery, and its persistence may impair normal organ function (Papalamprou et al., 2016; Phuengkham et al., 2018; Riley et al., 2019).

## Injectable Biomaterial Scaffolds for Immunotherapy

### Injectable Hydrogels for Immunotherapy

Injectable biomaterial scaffolds are transformable gel-like biomaterials that can be injected into the tumor location or resection site to produce a strong local or systemic antitumor immune response (Nguyen et al., 2020; Villard et al., 2019; Wang et al., 2019a).

A representative research designed alginate hydrogels which have the ability of in situ pores formation for the delivery of cytokine GM-CSF (a trigger to recruit and proliferate DCs) in a sustained manner (Hamilton, 2019). These macroporous alginate hydrogels can serve as a supportive scaffold for the infiltration of cells. In C57BL/6J female mice, the continuous release of GM-CSF from hydrogels resulted in the recruitment of a large number of cells into the scaffold. CD11b+ CD11c+ DCs occupied more than 90% of the cells that infiltrated the material at day five. This study may pave the way for the further promotion of high effective, therapeutic antigen-specific tolerogenic vaccines (Verbeke and Mooney, 2015). In the follow-up study, the authors explored the potential of such hydrogels for the delivery of microparticles or peptide antigens, resulting in the recruitment and activation of engineered immune cells (Verbeke et al., 2017).

Another research developed an alginate hydrogel to deliver two FDA-approved drugs, including celecoxib, a specific inhibitor of cycloxygenase-2 (COX2), and programmed death 1 (PD-1) monoclonal antibody. Compared with blank hydrogel treated mice, the co-delivery of celecoxib and anti-PD-1 group achieved a 90% suppression of tumors in the B16-F10 mice model, indicating significantly improved antitumor activities. Notably, 56% of the treated mice achieved complete regression of tumors after three months of follow-up. Then, the authors anglicized the T cell infiltration of tumor tissues. The results indicated that the co-delivery of dual agents increased the expression of IFN-γ-expressing CD4+ and CD8+ T cells by 5-6-fold, when compared to the blank hydrogel group. These outcomes, along with the decrease in Tregs and myeloid derived suppressor cells (MDSCs), demonstrate the regulatory role of the immunosuppressive tumor microenvironment. Furthermore, the expression of anti-angiogenic chemokines C-X-C motif ligand (CXCL) 9 and CXCL10 increased, and the expression of interleukin (IL)-1, IL-6 and cycloxygenase-2 (COX2) decreased, resulting in the suppression of the pro-tumor angiogenic and inflammatory microenvironment (Li Y. et al., 2016).

Song et al. designed PEGylated poly(L-valine) copolymers for the delivery of tumor cell lysates (TCL) and TLR3 agonist poly(I:C). This novel vaccine formulation aimed to recruit, activate and mature DCs through the continuous release of TCL and poly(I:C). The results demonstrated that this polypeptide hydrogel could sustainably release antigens or poly(I:C) for over seven days. In the melanoma mouse model, hydrogel formulations injected subcutaneously increased the percentage of migratory DCs in tumor-draining lymph nodes, and evoked a powerful cytotoxic T-lymphocyte immune response (Song et al., 2018).

In addition, a research developed a vaccine nodule that consisted of RADA16 peptide nanofibrous hydrogel, anti-PD-1 antibodies, DCs, and tumor cell lysates (TCL). After the subcutaneous injection into lymphoma mice, Gel-DC-TCL, with or without anti-PD-1 immunotherapy can improve the percentage of CD8+ IFN-γ+ T cells by 5-6 times, indicating the induction of powerful immune response of T cells (Yang et al., 2018).

**FIGURE 3:**
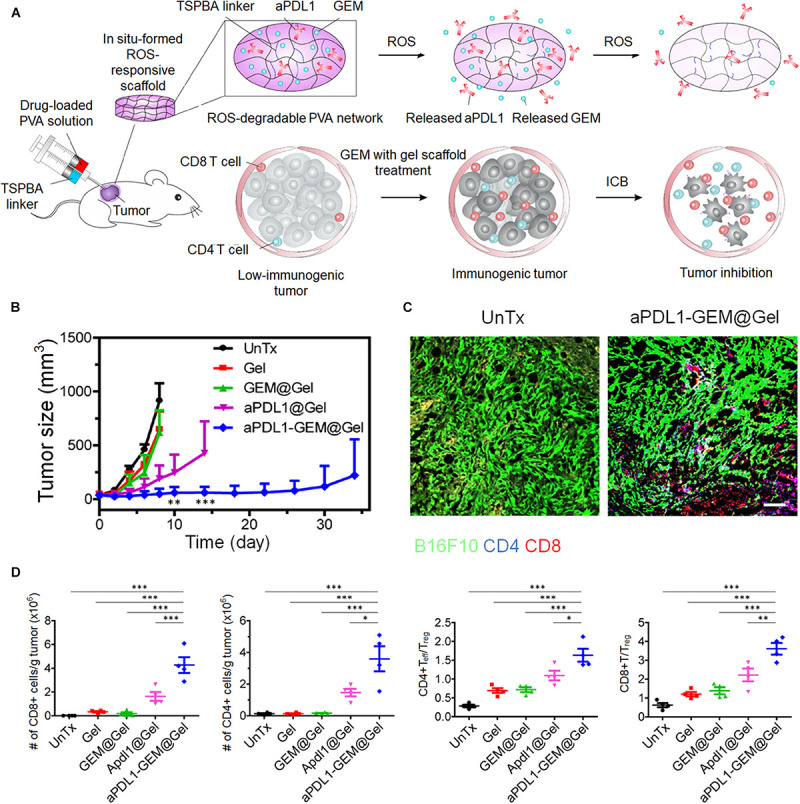
The ROS-responsive scaffold loaded with GEM and checkpoint inhibitor for chemoimmunotherapy. **(A)** ROS-degradable hydrogel scaffolds can achieve the co-delivery of GEM and aPDL1 into the tumor microenvironment for combination therapy. **(B)** The tumor growth curves of all groups. **(C)** The conditions of CD4+ and CD8+ T cell infiltration. Scale bar: 100 μm. **(D)** The numbers and ratios of immune cells in tumors under multiple treatments. Reproduced with permission from Wang C. et al. (2018).

As previously confirmed, the tumor microenvironment expressed abundant reactive oxygen species (ROS) to accelerate tumor progression (Daum et al., 2017; Ruan et al., 2018). Therefore, Wang et al. developed an in situ formed ROS-degradable hydrogel scaffold, which can achieve sustained release inside the microenvironment of tumors, for the localized delivery of GEM and anti-PD-L1 blocking antibody (aPDL1). The ROS-degradable hydrogel scaffold was synthesized by crosslinking poly (vinyl alcohol) (PVA) with a ROS-labile linker. In the B16F10 and 4T1 mice model, mice administrated with aPDL1-GEM@Gel demonstrated obvious tumor suppression effects. Furthermore, 50% of mice survived for more than 60 days after injected the aPDL1-GEM@Gel. However, mice in all control groups were sacrificed after two months. In order to further evaluate the immune regulation, the authors used immunofluorescence and flow cytometry to analyze the tumors on the 10th day after injection. The results revealed that aPDL1-GEM@Gel can trigger a powerful anti-tumor immune response induced by T cells. Remarkably, the hydrogel scaffolds can not only be used as a warehouse for the regulated disperse of therapeutic drugs, but also as a ROS cleaner to enhance the immunogenic phenotypes. This approach might provide a promising method for the treatment of low-immunogenic tumors (Figure 3; Wang C. et al., 2018).

Luo et al. developed a self-assembling hydrogel of a D-tetra-peptide (D-gel) to promote the clinical efficacy of vaccine adjuvant. In the B16-OVA mice model, D-gel loaded with OVA achieved 50% elimination of the tumors on contrast to other groups and had no weight loss of mice, demonstrating its antitumor potential and biocompatibility. In order to analysis the potential application of D-gel for complex antigens, the authors used X-ray irradiated E.G7 tumor cells and 4T1 tumor cells to serve as tumor antigens (Wang et al., 2019b). The results indicated that D-gel was a promising vaccine adjuvant for various kind of antigens. Furthermore, the authors test the immune response of D-gel loaded with X-ray irradiated E.G7 tumor cells. The results showed that CD8+ IFN-γ+ T cells proliferated obviously in the tumor microenvironment. Overall, this original strategy of vaccine adjuvant can act as a promising alternative for cancer immunotherapy (Luo et al., 2017).

Han et al. demonstrated a phospholipid-based phase separation hydrogel (PPSG) to deliver the antigen OVA and the adjuvant CpG-ODN. DCs were mass recruited to the injection position at 7 days after administrated with PPSG formulation, indicating the powerful and long-lasting immune regulation ability of this hydrogel tumor vaccine. Besides, with the degradation of hydrogel and the release of payloads, no immune regulatory effects were found at day 28. Furthermore, the authors tested the memory immune responses of PPSG formulation and indicated memory antibody responses and powerful memory T cell production (Han et al., 2016).

Hydrogel also showed obvious advantages in cytokine delivery. A representative study developed a hyaluronic acid tyramine (HA-Tyr) hydrogel for the delivery of IFN-α and sorafenib to treat renal cell carcinoma (RCC). In human RCC cells xenografted mice model, the combination of IFN-α and sorafenib demonstrated the best anti-tumor effect compared with other groups, indicating the synergistic effect of these two payloads. However, no statistical difference was discovered between the co-delivery group and the sorafenib group. In spite of this, the prolonged half-life of IFN-α and the achieved tumor suppression effect still cannot be ignored (Ueda et al., 2016).

Leach et al. described a peptide hydrogel based on positive charge Multi-Domain Peptide (MDP) for the delivery and controlled release of cyclic dinucleotides (CDNs). Notably, significant tumor inhibition effect was found in mice oral tumor model through a single injection of the hydrogel formulation at 3 days after the tumors were planted. The results showed that 60% of the mice was achieved adaptive immunity. No tumor growth was found with the treated of the hydrogel formulation after secondary tumor implantation. Besides, the controlled release time was demonstrated to be at least 7 days to maintain the CDN concentration around the injected site (Leach et al., 2018).

### Injectable Mesoporous Silica Rods for *in situ* Tumor Vaccine

Kim et al. developed a promising approach for in situ tumor vaccine based on self-assembled mesoporous silica rods (MSRs) of a high aspect ratio. MSRs have been extensively applied because of the sustained delivery of drugs due to its high porosity, extended superficial area, and biocompatibility (Chang et al., 2018; Wang Z et al., 2018). After administration in mice, this system can nonspecifically assemble into pore structures, allowing for the long and controlled release of payloads. The authors compared spontaneously assembled MSR structures to the randomly assembled matchsticks, resulting in the build of 3D porous structures to host immune cells, and the release of embedded immune agents. The agents in MSRs can recruit and programme host immune cells, and induce these cells to interact with other kinds of immune cells. MSR-based scaffolds, including OVA, GM-CSF and CpG-ODN, were further researched for their role as vaccines. After analyzing the cell recruitment of these scaffolds, the investigators found that the number of cells that remained in the vaccine MSR scaffolds was 6.5 folds higher than in blank MSR scaffolds at day seven. Then, the investigators analyzed their cell types, and indicated that CD11c+ DCs occupied 10% of the recruited cells. High levels of GM-CSF were detected in tissues between 1 mm and 3 mm from the injection site, demonstrating the release of GM-CSF in vivo. In addition, systemic TH1 and TH2 serum antibody and cytotoxic T cells were also significantly enhanced. These results show that injectable MSRs can be used as a multifunctional vaccine platform to regulate the function of immune cells, and trigger adaptive immune response (Kim et al., 2015).

Another research reported a simple approach to promote antigen immunogenicity through the combination of polyethyleneimine (PEI) with a mesoporous silica micro-rod (MSR) vaccine. The MSR-PEI vaccine was demonstrated to enhance the concentration and activation of DCs, and the immune response of T cells effectively, resulting in more effective humoral responses and tumor prevention effect compared to traditional vaccine formulation. Surprisingly, approximately 80% of mice with large established TC-1 tumors achieved completely tumor elimination through single injection of the MSC-PEI vaccine based on the E7 peptide. It is worth noting that the MSR-PEI vaccine can eradicate the established lung metastases when immunized with the B16F10 or CT26 neoantigen library. Overall, this research demonstrated a potentially modular strategy to promote the efficacy of immunotherapy. The vaccine can rapidly assemble to drive immune responses against the cancer-specific mutation pool, and be synergistic with other immunotherapies, achieving the vaccination of personalized vaccine (Li A.W. et al., 2018).

**FIGURE 4:**
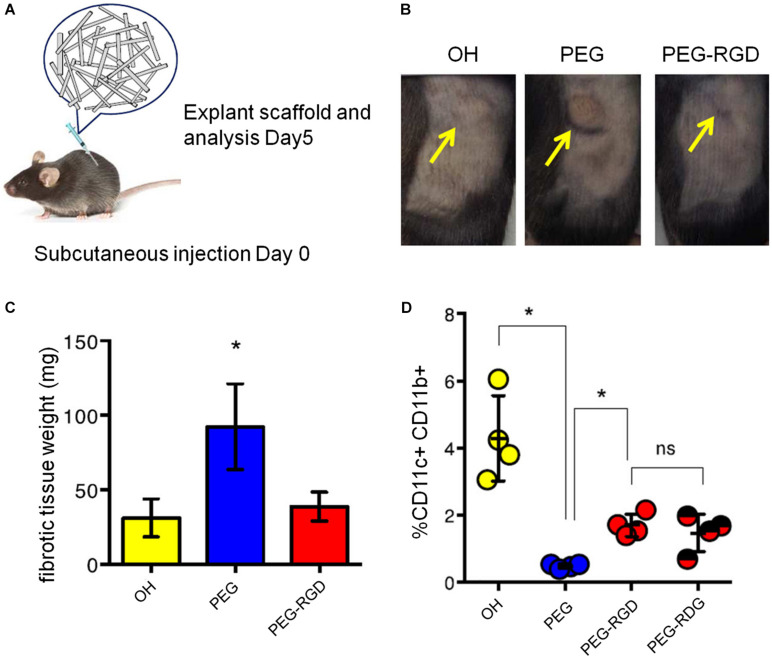
Comparison of various kinds of surface modified MSR scaffolds in therapeutic effect. **(A)** The experimental procedure of treatment. **(B)** Mice after injected with different modified MSR scaffolds, including unmodified MSRs (OH), PEG modified MSRs (PEG) and Poly (ethylene glycol) and integrin-binding ligand Arg-Gly-Asp modified MSRs (PEG-RGD). **(C)** Weight of the fibrous capsules. **(D)** Percentage of CD11c+ and CD11b+ cells in the scaffolds. (Reproduced with permission from Li W.A. et al. (2016). Unmodified MSRs, PEG MSRs, PEG-RGD MSRs and PEG-RDG MSRs were injected subcutaneously into the flank of mice. Poly (ethylene glycol) (PEG) and integrin-binding ligand Arg-Gly-Asp (RGD).

To explore the effects of surface modification of MSRs in inducing and regulating the immune system, a further study modified the Poly (ethylene glycol) (PEG) and integrin-binding ligand Arg-Gly-Asp (RGD) with MSR scaffolds. The results revealed that PEG modification increased the expression of the BMDC activation marker and IL-1β. The infiltration of innate immune cells was also increased. Meanwhile, the peptide-modified MSRs presented a reduction in inflammation, when compared with PEG MSRs. Besides, the authors investigated the weight of fibrous capsules in the surrounding of the scaffold, indicating PEG scaffold was the heaviest in all groups. These results indicate that surface modulation of these scaffolds can adjust the infiltration of immune cells, providing a promising alternative for the progress of new material-based vaccines (Figure 4; Li W.A. et al., 2016).

As mentioned above, the implantable biomaterial scaffolds can not only serve as durable warehouses for storing drugs or immune cells, but also remain at the site of the implantation for a long time to maintain the ability of antigen presentation, regulate cell transport, and perform a variety of else functions (Amir Afshar and Ghaee, 2016; Chen et al., 2015; Sinha et al., 2019; Zhang et al., 2018). Furthermore, implantable scaffolds need a small invasive surgical procedure to implant into the resected or the subcutaneous tissue space. Thus, such scaffolds are unable to be placed in volume-sensitive areas that cannot be reached by surgery, and the continued presence may impair normal organ function (Riley et al., 2019). Compared with implantable biomaterial scaffolds, injectable scaffolds are simpler to operate, and are less invasive, avoiding unwanted tissue injure and complications correlated to inflammatory response to wound (Norouzi et al., 2016; Shen et al., 2017). These scaffolds can reach anywhere through the needle, thereby avoiding unnecessary tissue damage (Zheng Y et al., 2019). Besides, due to the viscoelastic properties of injectable biomaterial scaffolds, they are able to flow to occupy discrete places (Hu et al., 2017; Leach et al., 2019). However, injectable scaffolds need sufficient fluidity to pass through the needle, which limits the use of many ideal materials (Lei and Tang, 2019; Qi et al., 2018). Besides, the selected biomaterials must have the ability to form a liquid or gel to pass through the needle, resulting in limitations of the types of materials and ingredients that can be used. Many suitable and excellent materials cannot meet the conditions required for injection, which seriously affects the application space of injectable materials and cannot realize complex three-dimensional structures. In short, for injectable and implantable scaffolds, as long as they are used flexibly according to the application environment and conditions, they can all play their respective advantages.

## Conclusion and Perspectives

The research on the combination of biomaterials and immunotherapy are rapidly advancing to break the scientific barriers, and overcoming present immunotherapy deficiencies (Eppler and Jewell, 2020; Scheetz et al., 2019). Among these, implantable and injectable biomaterial scaffolds present a promising potential for biomaterial delivery systems (Zhao et al., 2019). The low accumulation and expansion of tumor-reactive lymphocytes at the tumor sites, and the complex procedure significantly impact the therapeutic effect of adoptive cellular immunotherapy (Beatty and Gladney, 2015). However, the allowable dose confined by the autoimmunity still the main limitation of the application of checkpoint inhibitors, cytokines and agonistic antibodies therapies in clinical practice (Sanmamed and Chen, 2018; Szeto and Finley, 2019). Furthermore, the complex tumor microenvironment of solid tumors is another reason for the ineffective immunotherapy (Xie et al., 2019). The development of delivery strategies based on biomaterials improve the effect of immunotherapy to some extent, such as the recruiting and regulating lymphocytes in situ, reducing the degradation of therapeutic substances, improving the targeting capability of drugs, helping overcoming the physical barrier, reducing systemic side effects, and achieving persistent release. Other types of biomaterials, such as nanoparticles have also contributed greatly to the development of immunotherapy. Different kinds biomaterials make up for various defects of immunotherapy by virtue of their own advantages, which expands the application range of immunotherapy towards tumors and provides the possibility of personalized tumor treatment. Moreover, many studies have demonstrated that the combination of immunotherapy with other traditional therapies, including chemotherapy, phototherapy and radiotherapy, can produce synergistic effect and improved therapeutic efficacy of malignancies. Traditional therapies can not only eliminate tumor cells directly, but also mediate the immune process through inducing immune cell death of tumor cells. To date, an increasing number of successful attempts have indicated the potential of combining immunotherapy with other traditional therapies.

However, many problems still need to be solved before the widely applicable of immunotherapy to patients. Future research should develop new delivery technologies to achieve more efficient and secure delivery approaches of immunotherapy agents, when compared to present delivery strategies. New delivery strategies should also proliferate and engineer immune cell therapies ex vivo. Since the expansion rate of T cells is not ideal at present in cell therapy, biomaterials should also be considered to promote the proliferation and function of immune cells ex vivo, so as to enhance T cell delivery by increasing the migration to target tissues of reduced off-target effects in further studies. In addition to optimizing delivery, future studies should also investigate externally or internally induced delivery technologies. Therapeutic agents and engineered immune cells can be induced on demand to mediate the immune response in these systems, resulting in the reduction in off-tissue effects. Although significant progress has been made in immunotherapy, the design of delivery strategies in the area remains at its nascent stage. Furthermore, fundamental studies on biomaterial immune cell interactions are needed for the development of new delivery technologies, and the active directing of immune responses.

Although the immunotherapy of cancer is developing continuously has made long term progress, the multiple delivery systems for this field still have many limitations. The examples of implantable and injectable biomaterial scaffolds in our review not only provide methods to enhance the immunotherapy, but discuss the way to overcome the inherent heterogeneity of tumors. The payloads can be specifically selected according to the characteristics of different patients, which can improve the comprehensiveness and potential efficacy of immunotherapy. This review summarizes the combination of immunotherapy and biomaterials at the basic and application levels. We hope to made contributions for future innovations of cancer immunotherapy.

## Author Contributions

JL and YL wrote the manuscript. JL, YL, BL, YX, and HW revised the manuscript. CF designed the work of review and revised the manuscript. All authors contributed to the article and approved the submitted version.

## Conflict of Interest

The authors declare that the research was conducted in the absence of any commercial or financial relationships that could be construed as a potential conflict of interest.

## References

[B1] Ahn Y. H. Ren L. Kim S. M. Seo S.-H. Jung C. R. Kim D. S. (2020). A three-dimensional hyaluronic acid-based niche enhances the therapeutic efficacy of human natural killer cell-based cancer immunotherapy. *Biomaterials* 247:119960. 10.1016/j.biomaterials.2020.119960 32278822

[B2] Ali O. A. Huebsch N. Cao L. Dranoff G. Mooney D. J. (2009). Infection-mimicking materials to program dendritic cells in situ. *Nat. Mater.* 8 151–158. 10.1038/nmat2357 19136947 PMC2684978

[B3] Amir Afshar H. Ghaee A. (2016). Preparation of aminated chitosan/alginate scaffold containing halloysite nanotubes with improved cell attachment. *Carbohydrate Polym.* 151 1120–1131. 10.1016/j.carbpol.2016.06.063 27474663

[B4] Aurisicchio L. Pallocca M. Ciliberto G. Palombo F. (2018). The perfect personalized cancer therapy: cancer vaccines against neoantigens. *J. Exp. Clin. Cancer Res.* 37:86. 10.1186/s13046-018-0751-1 29678194 PMC5910567

[B5] Beatty G. L. Gladney W. L. (2015). Immune escape mechanisms as a guide for cancer Immunotherapy. *Clin. Cancer Res.* 21:687. 10.1158/1078-0432.CCR-14-1860 25501578 PMC4334715

[B6] Bersani F. Lee J. Yu M. Morris R. Desai R. Ramaswamy S. (2014). Bioengineered implantable scaffolds as a tool to study stromal-derived factors in metastatic cancer models. *Cancer Res.* 74:7229. 10.1158/0008-5472.CAN-14-1809 25339351 PMC4267901

[B7] Butterfield L. H. (2015). Cancer vaccines. *Br. Med. J.* 350:h988. 10.1136/bmj.h988 25904595 PMC4707521

[B8] Chang Z. M. Wang Z. Shao D. Yue J. Xing H. Li L. (2018). Shape engineering boosts magnetic mesoporous silica nanoparticle-based Isolation and detection of circulating tumor cells. *ACS Appl. Mater. Interf.* 10 10656–10663. 10.1021/acsami.7b19325 29468874

[B9] Chen C.-Y. Ke C.-J. Yen K.-C. Hsieh H.-C. Sun J.-S. Lin F.-H. (2015). 3D porous calcium-Alginate scaffolds cell culture system improved human osteoblast cell clusters for cell therapy. *Theranostics* 5 643–655. 10.7150/thno.11372 25825603 PMC4377732

[B10] Chen J. Jiang Z. Xu W. Sun T. Zhuang X. Ding J. (2020). Spatiotemporally targeted nanomedicine overcomes hypoxia-induced drug resistance of tumor cells after disrupting Neovasculature. *Nano Lett.* 20 6191–6198. 10.1021/acs.nanolett.0c02515 32697585

[B11] Chen Q. Chen M. Liu Z. (2019). Local biomaterials-assisted cancer immunotherapy to trigger systemic antitumor responses. *Chem. Soc. Rev.* 48 5506–5526. 10.1039/C9CS00271E 31589233

[B12] Chew S. A. Danti S. (2017). Biomaterial-based implantable devices for cancer therapy. *Adv. Healthcare Mater.* 6:1600766. 10.1002/adhm.201600766 27886461

[B13] Chung L. Maestas D. R. Housseau F. Elisseeff J. H. (2017). Key players in the immune response to biomaterial scaffolds for regenerative medicine. *Adv. Drug Deliv. Rev.* 114 184–192. 10.1016/j.addr.2017.07.006 28712923

[B14] Daum S. Reshetnikov M. S. V. Sisa M. Dumych T. Lootsik M. D. Bilyy R. (2017). Lysosome-targeting amplifiers of reactive oxygen species as anticancer prodrugs. *Angew. Chem. Int. Edn.* 56 15545–15549. 10.1002/anie.201706585 28994179

[B15] Davis Z. B. Vallera D. A. Miller J. S. Felices M. (2017). Natural killer cells unleashed: checkpoint receptor blockade and BiKE/TriKE utilization in NK-mediated anti-tumor immunotherapy. *Semin. Immunol.* 31 64–75. 10.1016/j.smim.2017.07.011 28882429 PMC5632228

[B16] Ding J. Chen J. Gao L. Jiang Z. Zhang Y. Li M. (2019a). Engineered nanomedicines with enhanced tumor penetration. *Nano Today* 29:100800 10.1016/j.nantod.2019.100800

[B17] Ding J. Feng X. Jiang Z. Xu W. Guo H. Zhuang X. (2019b). Polymer-mediated penetration-independent cancer therapy. *Biomacromolecules* 20 4258–4271. 10.1021/acs.biomac.9b01263 31668061

[B18] Ding J. Zhang J. Li J. Li D. Xiao C. Xiao H. (2019c). Electrospun polymer biomaterials. *Prog. Polym. Sci.* 90 1–34. 10.1016/j.progpolymsci.2019.01.002

[B19] Eppler H. B. Jewell C. M. (2020). Biomaterials as tools to decode immunity. *Adv. Mater.* 32:1903367. 10.1002/adma.201903367 31782844 PMC7124992

[B20] Feng X. Li J. Zhang X. Liu T. Ding J. Chen X. (2019a). Electrospun polymer micro/nanofibers as pharmaceutical repositories for healthcare. *J. Control. Release* 302 19–41. 10.1016/j.jconrel.2019.03.020 30922946

[B21] Feng X. Liu J. Xu W. Li G. Ding J. (2020). Tackling autoimmunity with nanomedicines. *Nanomedicine* 15 1585–1597. 10.2217/nnm-2020-0102 32669025

[B22] Feng X. Xu W. Li Z. Song W. Ding J. Chen X. (2019b). Immunomodulatory Nanosystems. *Adv. Sci.* 6:1900101. 10.1002/advs.201900101 31508270 PMC6724480

[B23] Guillerey C. Huntington N. D. Smyth M. J. (2016). Targeting natural killer cells in cancer immunotherapy. *Nat. Immunol.* 17 1025–1036. 10.1038/ni.3518 27540992

[B24] Guo H. Li F. Qiu H. Xu W. Li P. Hou Y. (2020). Synergistically enhanced mucoadhesive and penetrable polypeptide nanogel for efficient drug delivery to orthotopic bladder cancer. *Research* 2020 1–14. 10.34133/2020/8970135 32832909 PMC7420878

[B25] Hamilton J. A. (2019). GM-CSF in inflammation. *J. Exp. Med.* 217:e20190945. 10.1084/jem.20190945 31611249 PMC7037240

[B26] Han L. Xue J. Wang L. Peng K. Zhang Z. Gong T. (2016). An injectable, low-toxicity phospholipid-based phase separation gel that induces strong and persistent immune responses in mice. *Biomaterials* 105 185–194. 10.1016/j.biomaterials.2016.08.007 27522253

[B27] Hu C. Liu X. Ran W. Meng J. Zhai Y. Zhang P. (2017). Regulating cancer associated fibroblasts with losartan-loaded injectable peptide hydrogel to potentiate chemotherapy in inhibiting growth and lung metastasis of triple negative breast cancer. *Biomaterials* 144 60–72. 10.1016/j.biomaterials.2017.08.009 28823844

[B28] Kim J. Li W. A. Choi Y. Lewin S. A. Verbeke C. S. Dranoff G. (2015). Injectable, spontaneously assembling, inorganic scaffolds modulate immune cells in vivo and increase vaccine efficacy. *Nat. Biotechnol.* 33 64–72. 10.1038/nbt.3071 25485616 PMC4318563

[B29] Koshy S. T. Mooney D. J. (2016). Biomaterials for enhancing anti-cancer immunity. *Curr. Opin. Biotechnol.* 40 1–8. 10.1016/j.copbio.2016.02.001 26896596 PMC4975655

[B30] Leach D. G. Dharmaraj N. Piotrowski S. L. Lopez-Silva T. L. Lei Y. L. Sikora A. G. (2018). STINGel: controlled release of a cyclic dinucleotide for enhanced cancer immunotherapy. *Biomaterials* 163 67–75. 10.1016/j.biomaterials.2018.01.035 29454236 PMC5840037

[B31] Leach D. G. Young S. Hartgerink J. D. (2019). Advances in immunotherapy delivery from implantable and injectable biomaterials. *Acta Biomater.* 88 15–31. 10.1016/j.actbio.2019.02.016 30771535 PMC6632081

[B32] Lei K. Tang L. (2019). Surgery-free injectable macroscale biomaterials for local cancer immunotherapy. *Biomater. Sci.* 7 733–749. 10.1039/C8BM01470A 30637428

[B33] Li A. W. Sobral M. C. Badrinath S. Choi Y. Graveline A. Stafford A. G. (2018). A facile approach to enhance antigen response for personalized cancer vaccination. *Nat. Mater.* 17 528–534. 10.1038/s41563-018-0028-2 29507416 PMC5970019

[B34] Li S. Feng X. Wang J. He L. Wang C. Ding J. (2018). Polymer nanoparticles as adjuvants in cancer immunotherapy. *Nano Res.* 11 5769–5786. 10.1007/s12274-018-2124-7

[B35] Li S. Feng X. Wang J. Xu W. Islam M. A. Sun T. (2019). Multiantigenic nanoformulations activate anticancer immunity depending on size. *Adv. Funct. Mater.* 29:1903391 10.1002/adfm.201903391

[B36] Li W. A. Lu B. Y. Gu L. Choi Y. Kim J. Mooney D. J. (2016). The effect of surface modification of mesoporous silica micro-rod scaffold on immune cell activation and infiltration. *Biomaterials* 83 249–256. 10.1016/j.biomaterials.2016.01.026 26784009 PMC4754159

[B37] Li Y. Fang M. Zhang J. Wang J. Song Y. Shi J. (2016). Hydrogel dual delivered celecoxib and anti-PD-1 synergistically improve antitumor immunity. *OncoImmunology* 5:e1074374. 10.1080/2162402X.2015.1074374 27057439 PMC4801446

[B38] Lohmueller J. Finn O. J. (2017). Current modalities in cancer immunotherapy: immunomodulatory antibodies. CARs and vaccines. *Pharmacol. Ther.* 178 31–47. 10.1016/j.pharmthera.2017.03.008 28322974 PMC5600680

[B39] López-Soto A. Gonzalez S. Folgueras A. R. (2017). IFN signaling and ICB resistance: time is on tumor’s side. *Trends Cancer* 3 161–163. 10.1016/j.trecan.2017.01.004 28718428

[B40] Luo Z. Wu Q. Yang C. Wang H. He T. Wang Y. (2017). A Powerful CD8+ T-cell stimulating D-Tetra-peptide hydrogel as a very promising vaccine adjuvant. *Adv. Mater.* 29:1601776. 10.1002/adma.201601776 27859662

[B41] Majzner R. G. Mackall C. L. (2018). Tumor antigen escape from CAR T-cell therapy. *Cancer Discov.* 8:1219. 10.1158/2159-8290.CD-18-0442 30135176

[B42] Mouthuy P. A. Snelling S. J. B. Dakin S. G. Milković L. Gašparović A. Č Carr A. J. (2016). Biocompatibility of implantable materials: an oxidative stress viewpoint. *Biomaterials* 109 55–68. 10.1016/j.biomaterials.2016.09.010 27669498

[B43] Neelapu S. S. Tummala S. Kebriaei P. Wierda W. Gutierrez C. Locke F. L. (2018). Chimeric antigen receptor T-cell therapy — assessment and management of toxicities. *Nat. Rev. Clin. Oncol.* 15 47–62. 10.1038/nrclinonc.2017.148 28925994 PMC6733403

[B44] Nguyen T. L. Yin Y. Choi Y. Jeong J. H. Kim J. (2020). Enhanced Cancer DNA vaccine via direct transfection to host dendritic cells recruited in Injectable Scaffolds. *ACS Nano* 14 11623–11636. 10.1021/acsnano.0c04188 32808762

[B45] Norouzi M. Nazari B. Miller D. W. (2016). Injectable hydrogel-based drug delivery systems for local cancer therapy. *Drug Discov. Today* 21 1835–1849. 10.1016/j.drudis.2016.07.006 27423369

[B46] Papalamprou A. Chang C. W. Vapniarsky N. Clark A. Walker N. Griffiths L. G. (2016). Xenogeneic cardiac extracellular matrix scaffolds with or without seeded mesenchymal stem cells exhibit distinct in vivo immunosuppressive and regenerative properties. *Acta Biomater.* 45 155–168. 10.1016/j.actbio.2016.07.032 27445086

[B47] Pelaez F. Manuchehrabadi N. Roy P. Natesan H. Wang Y. Racila E. (2018). Biomaterial scaffolds for non-invasive focal hyperthermia as a potential tool to ablate metastatic cancer cells. *Biomaterials* 166 27–37. 10.1016/j.biomaterials.2018.02.048 29533788

[B48] Phuengkham H. Song C. Um S. H. Lim Y. T. (2018). Implantable synthetic immune niche for spatiotemporal modulation of tumor-derived immunosuppression and systemic antitumor immunity: postoperative immunotherapy. *Adv. Mater.* 30:1706719. 10.1002/adma.201706719 29572968

[B49] Postow M. A. Callahan M. K. Wolchok J. D. (2015). Immune checkpoint blockade in cancer therapy. *J. Clin. Oncol.* 33 1974–1982. 10.1200/JCO.2014.59.4358 25605845 PMC4980573

[B50] Qi Y. Min H. Mujeeb A. Zhang Y. Han X. Zhao X. (2018). Injectable hexapeptide hydrogel for localized chemotherapy prevents breast cancer recurrence. *ACS Appl. Mater. Interf.* 10 6972–6981. 10.1021/acsami.7b19258 29409316

[B51] Qiu H. Guo H. Li D. Hou Y. Kuang T. Ding J. (2020). Intravesical hydrogels as drug reservoirs. *Trends Biotechnol.* 38 579–583. 10.1016/j.tibtech.2019.12.012 31926600

[B52] Rao S. S. Bushnell G. G. Azarin S. M. Spicer G. Aguado B. A. Stoehr J. R. (2016). Enhanced survival with implantable scaffolds that capture metastatic breast cancer cells andlt;emandgt;In Vivoandlt;/emandgt. *Cancer Res.* 76:5209. 10.1158/0008-5472.CAN-15-2106 27635043 PMC5027988

[B53] Riley R. S. June C. H. Langer R. Mitchell M. J. (2019). Delivery technologies for cancer immunotherapy. *Nat. Rev. Drug Discov.* 18 175–196. 10.1038/s41573-018-0006-z 30622344 PMC6410566

[B54] Rooney C. Sauer T. (2018). Modeling cytokine release syndrome. *Nat. Med.* 24 705–706. 10.1038/s41591-018-0068-9 29808004

[B55] Ruan C. Liu L. Wang Q. Chen X. Chen Q. Lu Y. (2018). Reactive oxygen species-biodegradable gene carrier for the targeting therapy of breast cancer. *ACS Appl. Mater. Interf.* 10 10398–10408. 10.1021/acsami.8b01712 29498264

[B56] Sabado R. L. Bhardwaj N. (2015). Dendritic-cell vaccines on the move. *Nature* 519 300–301. 10.1038/nature14211 25762139

[B57] Sahin U. Türeci Ö (2018). Personalized vaccines for cancer immunotherapy. *Science* 359:1355. 10.1126/science.aar7112 29567706

[B58] Sanchez-Correa B. Lopez-Sejas N. Duran E. Labella F. Alonso C. Solana R. (2019). Modulation of NK cells with checkpoint inhibitors in the context of cancer immunotherapy. *Cancer Immunol. Immunother.* 68 861–870. 10.1007/s00262-019-02336-6 30953117 PMC11028212

[B59] Sang W. Zhang Z. Dai Y. Chen X. (2019). Recent advances in nanomaterial-based synergistic combination cancer immunotherapy. *Chem. Soc. Rev.* 48 3771–3810. 10.1039/C8CS00896E 31165801

[B60] Sanmamed M. F. Chen L. (2018). A paradigm shift in cancer immunotherapy: from enhancement to normalization. *Cell* 175 313–326. 10.1016/j.cell.2018.09.035 30290139 PMC6538253

[B61] Saxena M. Bhardwaj N. (2018). Re-Emergence of Dendritic Cell Vaccines for Cancer Treatment. *Trends Cancer* 4 119–137. 10.1016/j.trecan.2017.12.007 29458962 PMC5823288

[B62] Scheetz L. Park K. S. Li Q. Lowenstein P. R. Castro M. G. Schwendeman A. (2019). Engineering patient-specific cancer immunotherapies. *Nat. Biomed. Eng.* 3 768–782. 10.1038/s41551-019-0436-x 31406259 PMC6783331

[B63] Seib F. P. Berry J. E. Shiozawa Y. Taichman R. S. Kaplan D. L. (2015). Tissue engineering a surrogate niche for metastatic cancer cells. *Biomaterials* 51 313–319. 10.1016/j.biomaterials.2015.01.076 25771021 PMC4367489

[B64] Shen W. Chen X. Luan J. Wang D. Yu L. Ding J. (2017). Sustained codelivery of cisplatin and paclitaxel via an injectable prodrug hydrogel for ovarian cancer treatment. *ACS Appl. Mater. Interf.* 9 40031–40046. 10.1021/acsami.7b11998 29131563

[B65] Shi J. Darrah E. Sims G. P. Mustelin T. Sampson K. Konig M. F. (2018). Affinity maturation shapes the function of agonistic antibodies to peptidylarginine deiminase type 4 in rheumatoid arthritis. *Ann. Rheum. Dis.* 77:141. 10.1136/annrheumdis-2017-211489 29070531 PMC5935255

[B66] Shimasaki N. Jain A. Campana D. (2020). NK cells for cancer immunotherapy. *Nat. Rev. Drug Discov.* 19 200–218. 10.1038/s41573-019-0052-1 31907401

[B67] Siegler E. L. Zhu Y. Wang P. Yang L. (2018). Off-the-Shelf CAR-NK Cells for Cancer Immunotherapy. *Cell Stem Cell* 23 160–161. 10.1016/j.stem.2018.07.007 30075127

[B68] Sinha A. Choi Y. Nguyen M. H. Nguyen T. L. Choi S. W. Kim J. (2019). A 3D macroporous alginate graphene scaffold with an extremely slow release of a loaded cargo for in situ long-term activation of Dendritic Cells. *Adv. Healthcare Mater.* 8:1800571. 10.1002/adhm.201800571 30680955

[B69] Smith T. T. Moffett H. F. Stephan S. B. Opel C. F. Dumigan A. G. Jiang X. (2017). Biopolymers codelivering engineered T cells and STING agonists can eliminate heterogeneous tumors. *J. Clin. Invest.* 127 2176–2191. 10.1172/JCI87624 28436934 PMC5451231

[B70] Song H. Huang P. Niu J. Shi G. Zhang C. Kong D. (2018). Injectable polypeptide hydrogel for dual-delivery of antigen and TLR3 agonist to modulate dendritic cells in vivo and enhance potent cytotoxic T-lymphocyte response against melanoma. *Biomaterials* 159 119–129. 10.1016/j.biomaterials.2018.01.004 29324304

[B71] Srinivasan V. M. Ferguson S. D. Lee S. Weathers S.-P. Kerrigan B. C. P. Heimberger A. B. (2017). Tumor vaccines for malignant gliomas. *Neurotherapeutics* 14 345–357. 10.1007/s13311-017-0522-2 28389997 PMC5398993

[B72] Stephan S. B. Taber A. M. Jileaeva I. Pegues E. P. Sentman C. L. Stephan M. T. (2015). Biopolymer implants enhance the efficacy of adoptive T-cell therapy. *Nat. Biotechnol.* 33 97–101. 10.1038/nbt.3104 25503382 PMC4289408

[B73] Sterner R. M. Sakemura R. Cox M. J. Yang N. Khadka R. H. Forsman C. L. (2019). GM-CSF inhibition reduces cytokine release syndrome and neuroinflammation but enhances CAR-T cell function in xenografts. *Blood* 133 697–709. 10.1182/blood-2018-10-881722 30463995 PMC6376281

[B74] Suzuki E. Kapoor V. Jassar A. S. Kaiser L. R. Albelda S. M. (2005). Gemcitabine Selectively Eliminates Splenic Gr-1andlt;supandgt;+andlt;/supandgt;/CD11bandlt;supandgt;+andlt;/supandgt; myeloid suppressor cells in tumor-bearing animals and enhances antitumor immune activity. *Clin. Cancer Res.* 11:6713. 10.1158/1078-0432.CCR-05-0883 16166452

[B75] Szeto G. L. Finley S. D. (2019). Integrative approaches to cancer immunotherapy. *Trends Cancer* 5 400–410. 10.1016/j.trecan.2019.05.010 31311655 PMC7467854

[B76] Tian Z. (2017). NK cells and immunotherapy. *Semin. Immunol.* 31 1–2. 10.1016/j.smim.2017.09.008 28985906

[B77] Ueda K. Akiba J. Ogasawara S. Todoroki K. Nakayama M. Sumi A. (2016). Growth inhibitory effect of an injectable hyaluronic acid–tyramine hydrogels incorporating human natural interferon-α and sorafenib on renal cell carcinoma cells. *Acta Biomater.* 29 103–111. 10.1016/j.actbio.2015.10.024 26481041

[B78] Verbeke C. S. Gordo S. Schubert D. A. Lewin S. A. Desai R. M. Dobbins J. (2017). Multicomponent injectable hydrogels for antigen-specific tolerogenic immune modulation. *Adv. Healthcare Mater.* 6:1600773. 10.1002/adhm.201600773 28116870 PMC5518671

[B79] Verbeke C. S. Mooney D. J. (2015). Injectable, pore-forming hydrogels for in vivo enrichment of immature Dendritic Cells. *Adv. Healthcare Mater.* 4 2677–2687. 10.1002/adhm.201500618 26474318 PMC4715727

[B80] Villard P. Rezaeeyazdi M. Colombani T. Joshi-Navare K. Rana D. Memic A. (2019). Autoclavable and injectable cryogels for biomedical applications. *Adv. Healthcare Mater.* 8:1900679. 10.1002/adhm.201900679 31348620

[B81] Walsh M. C. Lee J. Choi Y. (2015). Tumor necrosis factor receptor- associated factor 6 (TRAF6) regulation of development, function, and homeostasis of the immune system. *Immunol. Rev.* 266 72–92. 10.1111/imr.12302 26085208 PMC4799835

[B82] Wang C. Wang J. Zhang X. Yu S. Wen D. Hu Q. (2018). In situ formed reactive oxygen species–responsive scaffold with gemcitabine and checkpoint inhibitor for combination therapy. *Sci. Transl. Med.* 10:eaan3682. 10.1126/scitranslmed.aan3682 29467299

[B83] Wang J. Li Z. Wang Z. Yu Y. Li D. Li B. (2020). Nanomaterials for Combinational radio–immuno oncotherapy. *Adv. Funct. Mater.* 30:1910676 10.1002/adfm.201910676

[B84] Wang P. Zhao P. Dong S. Xu T. He X. Chen M. (2018). An albumin-binding polypeptide both targets cytotoxic t lymphocyte vaccines to lymph nodes and boosts vaccine presentation by dendritic cells. *Theranostics* 8 223–236. 10.7150/thno.21691 29290804 PMC5743471

[B85] Wang Y. Jiang Z. Xu W. Yang Y. Zhuang X. Ding J. (2019a). Chiral polypeptide thermogels induce controlled inflammatory response as potential immunoadjuvants. *ACS Appl. Mater. Interf.* 11 8725–8730. 10.1021/acsami.9b01872 30785721

[B86] Wang Y. Zenkoh J. Gerelchuluun A. Sun L. Cai S. Li X. (2019b). Administration of dendritic cells and anti-PD-1 antibody converts X-ray irradiated tumors into effective in situ vaccines. *Int. J. Radiat. Oncol. Biol. Phys.* 103 958–969. 10.1016/j.ijrobp.2018.11.019 30458232

[B87] Wang Z. Chang Z. Lu M. Shao D. Yue J. Yang D. (2018). Shape-controlled magnetic mesoporous silica nanoparticles for magnetically-mediated suicide gene therapy of hepatocellular carcinoma. *Biomaterials* 154 147–157. 10.1016/j.biomaterials.2017.10.047 29128843

[B88] Wang Z. Wu Z. Liu Y. Han W. (2017). New development in CAR-T cell therapy. *J. Hematol. Oncol.* 10:53. 10.1186/s13045-017-0423-1 28222796 PMC5320663

[B89] Wei J. Han X. Bo J. Han W. (2019). Target selection for CAR-T therapy. *J. Hematol. Oncol.* 12:62. 10.1186/s13045-019-0758-x 31221182 PMC6587237

[B90] Wilky B. A. (2019). Immune checkpoint inhibitors: the linchpins of modern immunotherapy. *Immunol. Rev.* 290 6–23. 10.1111/imr.12766 31355494

[B91] Xie Y. J. Dougan M. Jailkhani N. Ingram J. Fang T. Kummer L. (2019). Nanobody-based CAR T cells that target the tumor microenvironment inhibit the growth of solid tumors in immunocompetent mice. *Proc. Natl. Acad. Sci. U.S.A.* 116 7624. 10.1073/pnas.1817147116 30936321 PMC6475367

[B92] Xie Y.-Q. Wei L. Tang L. (2018). Immunoengineering with biomaterials for enhanced cancer immunotherapy. *WIREs Nanomed. Nanobiotechnol.* 10:e1506. 10.1002/wnan.1506 29333729

[B93] Yang L. Li A. Lei Q. Zhang Y. (2019). Tumor-intrinsic signaling pathways: key roles in the regulation of the immunosuppressive tumor microenvironment. *J. Hematol. Oncol.* 12:125. 10.1186/s13045-019-0804-8 31775797 PMC6880373

[B94] Yang P. Song H. Qin Y. Huang P. Zhang C. Kong D. (2018). Engineering dendritic-cell-based vaccines and PD-1 blockade in self-assembled peptide nanofibrous hydrogel to amplify antitumor T-cell immunity. *Nano Lett.* 18 4377–4385. 10.1021/acs.nanolett.8b01406 29932335

[B95] Yang Y. (2015). Cancer immunotherapy: harnessing the immune system to battle cancer. *J. Clin. Invest.* 125 3335–3337. 10.1172/JCI83871 26325031 PMC4588312

[B96] Youssef A. Hollister S. J. Dalton P. D. (2017). Additive manufacturing of polymer melts for implantable medical devices and scaffolds. *Biofabrication* 9:012002. 10.1088/1758-5090/aa5766 28245199

[B97] Zhang H. Dong S. Li Z. Feng X. Xu W. Tulinao C. M. S. (2020). Biointerface engineering nanoplatforms for cancer-targeted drug delivery. *Asian J. Pharm. Sci.* 15 397–415. 10.1016/j.ajps.2019.11.004 32952666 PMC7486517

[B98] Zhang L. Fang H. Zhang K. Yin J. (2018). Homologous sodium alginate/Chitosan-based scaffolds, but contrasting effect on stem cell shape and osteogenesis. *ACS Appl. Mater. Interf.* 10 6930–6941. 10.1021/acsami.7b18859 29392929

[B99] Zhang Y. Bush X. Yan B. Chen J. A. (2019). Gemcitabine nanoparticles promote antitumor immunity against melanoma. *Biomaterials* 189 48–59. 10.1016/j.biomaterials.2018.10.022 30388589 PMC6281175

[B100] Zhao Z. Zheng L. Chen W. Weng W. Song J. Ji J. (2019). Delivery strategies of cancer immunotherapy: recent advances and future perspectives. *J. Hematol. Oncol.* 12:126. 10.1186/s13045-019-0817-3 31779642 PMC6883629

[B101] Zheng P. Liu Y. Chen J. Xu W. Li G. Ding J. (2019). Targeted pH-responsive polyion complex micelle for controlled intracellular drug delivery. *Chin. Chem. Lett.* 31 1178–1182. 10.1016/j.cclet.2019.12.001

[B102] Zheng Y. Wang W. Zhao J. Wu C. Ye C. Huang M. (2019). Preparation of injectable temperature-sensitive chitosan-based hydrogel for combined hyperthermia and chemotherapy of colon cancer. *Carbohydr. Polym.* 222:115039. 10.1016/j.carbpol.2019.115039 31320053

[B103] Zippelius A. Schreiner J. Herzig P. Müller P. (2015). Induced PD-L1 expression mediates acquired resistance to agonistic Anti-CD40 Treatment. *Cancer Immunol. Res.* 3:236. 10.1158/2326-6066.CIR-14-0226 25623164

